# Imitation and recognition of facial emotions in autism: a computer vision approach

**DOI:** 10.1186/s13229-021-00430-0

**Published:** 2021-04-06

**Authors:** Hanna Drimalla, Irina Baskow, Behnoush Behnia, Stefan Roepke, Isabel Dziobek

**Affiliations:** 1 Department of Psychology, Humboldt-Universität zu Berlin, Unter den Linden 6, 10099 Berlin, Germany; 2 Clinical Psychology of Social Interaction, Berlin School of Mind and Brain, Humboldt-Universität zu Berlin, Unter den Linden 6, 10099 Berlin, Germany; 3 Digital Health Center, Hasso Plattner Institute, University of Potsdam, Am Neuen Palais 10, 14469 Potsdam, Germany; 4 Departement of Psychiatry and Psychotherapy, Charité-Universitätsmedizin Berlin, corporate member of Freie Universität Berlin and Humboldt-Universität zu Berlin, Campus Benjamin Franklin, Hindenburgdamm 30, 12203 Berlin, Deutschland; 5 Multimodal Behavior Processing, Faculty of Technology, Bielefeld University, Inspiration 1, 33619 Bielefeld, Germany

**Keywords:** Autism, Imitation, Facial expression, Emotion recognition, Automated analysis

## Abstract

**Background:**

Imitation of facial expressions plays an important role in social functioning. However, little is known about the quality of facial imitation in individuals with autism and its relationship with defining difficulties in emotion recognition.

**Methods:**

We investigated imitation and recognition of facial expressions in 37 individuals with autism spectrum conditions and 43 neurotypical controls. Using a novel computer-based face analysis, we measured instructed imitation of facial emotional expressions and related it to emotion recognition abilities.

**Results:**

Individuals with autism imitated facial expressions if instructed to do so, but their imitation was both slower and less precise than that of neurotypical individuals. In both groups, a more precise imitation scaled positively with participants’ accuracy of emotion recognition.

**Limitations:**

Given the study’s focus on adults with autism without intellectual impairment, it is unclear whether the results generalize to children with autism or individuals with intellectual disability. Further, the new automated facial analysis, despite being less intrusive than electromyography, might be less sensitive.

**Conclusions:**

Group differences in emotion recognition, imitation and their interrelationships highlight potential for treatment of social interaction problems in individuals with autism.

## Introduction

Facial expressions are an essential tool to communicate emotions non-verbally in social interactions [1]. Being able to understand as well as to generate these expressions is crucial to the exchange of inner states with others [2]. Impairments in reciprocal social communication and interaction are key diagnostic aspects of autism spectrum conditions (ASC) [3]. Although the diagnosis includes both the understanding *and* the generation of non-verbal signals, especially the latter as well as the association of the two has neither been fully understood nor investigated.

In the context of this question, especially the ability to generate facial expressions that match the facial expression of the interaction partner might play a crucial role. Neurotypical (NT) individuals (i.e. individuals without autism) tend to mimic facial expressions in social interactions automatically [4]. There is evidence that such spontaneous facial imitation, often referred to as mimicry, might help people to recognize emotions (e.g. [5–9]).

Accordingly, many specific interventions for patients with ASC involve teaching the voluntary imitation of other’s facial emotions (e.g. [10–12]). However, the actual benefit of imitation that is voluntarily produced remains unclear, especially in individuals with autism. Investigating the relationship between the voluntary imitation of facial expressions and the recognition of those expressions in autism seems promising. It may be a possible key to elucidate the expression and recognition deficits, understand their interaction and target them therapeutically.

Although many studies reported difficulties of individuals with autism to recognize emotions, results remain inconsistent regarding specific emotions [13–15]. A reason might be the low sensitivity of many tasks. This becomes apparent in studies with high-functioning individuals, i.e. individuals who show only a mild level of symptoms and an intelligence quotient of 70 or above [16]. Generally, mixed results may be due to differences in the individuals’ level of functioning, potential compensatory mechanisms and task demands [15].

Regarding imitation of non-verbal signs and its important role in social functioning, surprisingly little is known about the tendency to imitate facial expressions in individuals with ASC. Compared to healthy controls, there seem to be differences in spontaneous imitation of facial expressions [17, 18], voluntary imitation, however, seems to be grossly unimpaired [18–20]. The limited evidence for aberrant voluntary imitation might be explained by ceiling effects [21] as most studies focused on the occurrence of imitation, ignoring its quality. However, the voluntary facial imitation capacity of individuals with ASC appears to differ from neurotypicals’ regarding quality [22] as well as timing [23]. A recent meta-analysis [24] summarized a variety of differences in how people with ASC express facial emotion expressions. However, the authors pointed out that the strength of the group differences may be overestimated due to confounding effects of age or intellectual functioning. In conclusion, the exact nature of facial imitation in adults with autism and without intellectual impairment has not yet been fully understood and deserves to be investigated further.

Although most studies investigated voluntary facial imitation of individuals with autism in the context of emotion recognition paradigms, the performance in both areas has not been linked in those studies (e.g. [18, 23]). One reason might be that the measures of emotion recognition as well as of imitation performance used in those studies were not sensitive enough, as they used easy-to-recognize emotions and measured only occurrence or speed but not the precision of imitation.

So far, most studies investigating the expression of facial emotions have either deployed electromyography, which has been reported as obtrusive, were limited to very few muscles, or have used time-costly coding of video-recorded expressions by observers. Due to current advances in image and video classification [25, 26], computer-based facial expression analysis offers new possibilities to measure facial expressions. This analysis classifies the purely visual input of facial features and facial motion into abstract classes [27]. Unlike electromyography, a computer-based analysis is neither expensive nor intrusive. It allows measurement of facial expression without physical contact with the participant (e.g. when applying the EMG electrodes). This is especially relevant in studies including individuals with autism, as touch is often perceived as aversive, might induce irritation and thus introduce confounds. A study on detection of autism diagnosis successfully classified individuals with autism based on their automatically analysed facial expressions [28]. Another recent study [29] analysed the spontaneous production of facial expressions using automated facial expression analysis software and related its relationships to alexithymia. Both studies clearly showed the value of automatic computer-based approaches.

Taken together, the relationship between recognition and imitation of facial expressions lacks rigorous investigation in adults with ASC and without intellectual impairment. A better understanding of the nature of both phenomena, as well as their association, might help to target the social struggle of individuals with autism. This study, therefore, seeks to examine the voluntary facial imitation capacity of individuals with and without autism in an emotion recognition paradigm. First, we expect to replicate the previously described emotion recognition deficit in individuals with ASC. Second, we assume quantitative as well as qualitative differences in facial imitation in individuals with autism. Third, we aim to elucidate the relationship between facial imitation and emotion recognition—especially for ASC.

### Methods

#### Participants

Thirty-seven adults with ASC (18 female, mean age = 36.89, range 22–62) and forty-three NT individuals (22 female; mean age = 33.14, range 18–49) with no self-reported history of psychiatric or neurological disorders participated in the study. Three participants had been excluded previously because of not matching the inclusion criteria. The remaining sample size of 80 exceeded the required sample size of 67 estimated by a statistical power analysis (evaluated for whole sample bivariate one-tailed correlations with power = 0.80, *α* = 0*.*05 and a medium effect size *ρ* = 0*.*30). Participants from the ASC group were recruited through the autism outpatient clinic of the Charité – Universitätsmedizin Berlin. All of the participants were diagnosed according to ICD-10 criteria for Asperger syndrome or atypical autism or childhood autism [30].

The diagnostic procedure included the Autism Diagnostic Observation Schedule (*n* = 36; ADOS-2; due to readability, the authors use the term ADOS; the group’s raw algorithm total score can be found in Table 1; all analyses were calculated on the social domain score of ADOS-2; [31]) and the Autism Diagnostic Interview-Revised (*n* = 22, ADI-R, diagnostic algorithm total score; [32]) if parental informants were available.

**Table 1:** Demographic and diagnostic information for participants with ASC and NT individuals

Demographic and diagnostic measures	ASC group			NT group		
	N	M	SD	N	M	SD
Age	37	36.89	10.44	43	33.14	8.5
Sex (female/male)	18:19	–	–	22:21	–	–
Verbal intelligence (WST)	35	112.29	11.15	42	109.19	9.06
Autistic traits (AQ)	35	37.54	5.74	41	14.49	5.82
ADOS (raw algorithm total score)	36	9.19	3.28	–	–	–
ADI-R (total score)	22	29.59	14.80	–	–	–

WST = Wortschatztest, a German vocabulary test to measure verbal intelligence; AQ = Autism-Spectrum Quotient; ADOS-2 = Autism Diagnostic Observation Schedule 2; ADI-R = Autism Diagnostic Interview-Revised. For ADI-R, an autism diagnosis is indicated when scores in all three behavioural areas meet the cut-off scores (social interaction: 10, communication and language: 8 and restricted and repetitive behaviours: 3). For ADOS-2, the cut-off for an “autism spectrum” diagnosis is 7 and the cut-off for an “autism” diagnosis is 10

Exclusion criteria were current antipsychotic and anticonvulsant medication, comorbid neurological disorders or age over 65 years to avoid possible confounding age-related neurodegeneration. Furthermore, high German language proficiency was demanded that was assessed through a German vocabulary test (Wortschatztest (WST), [33]). A further exclusion criterion was the use of any medical treatments (e.g. benzodiazepines) that could have an impact on the cognitive abilities of the participants. In addition, in the control group, any history of psychiatric disorder led to exclusion.

### Procedure

The experiments were conducted in a laboratory with constant lighting conditions. Participants were asked to engage in an emotion recognition and imitation task. During the experiment, the participants’ faces were recorded with a webcam with a rate of 30 frames per second and a resolution of 640 × 480 pixels. An effort was made to disguise the aim of the video recording so that participants would not concentrate on their facial movements: the experimenter told the participants that the webcam was only placed to monitor their attention level. The video recordings of all participants were checked individually and were, in case the instructions were not followed correctly, excluded.

### Measures

#### Emotion recognition

**Fig. 1:**
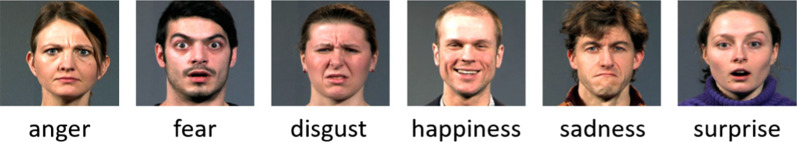
Example pictures of BERT for each emotion

The Berlin Emotion Recognition Test (BERT) [34] is a computer-based task for sensitively assessing emotion recognition. The test consists of a total of 48 photographs of facial expressions of professional actors displaying one of the six basic emotions ([35]; for stimulus production see [36]). The face is centred in front of a dark grey background. There are eight pictures per emotion, and each is expressed by four different female and four different male actors (see Fig. 1 for an example picture for each emotion). Below each picture, two emotional words are presented, and the participant is asked how the person is feeling. Only one of the two possible options correctly describes the emotion expressed. The emotion recognition score is the percentage of correct answers. The position of the correct answer, as well as the order of the picture, is randomized.

**Fig. 2:**
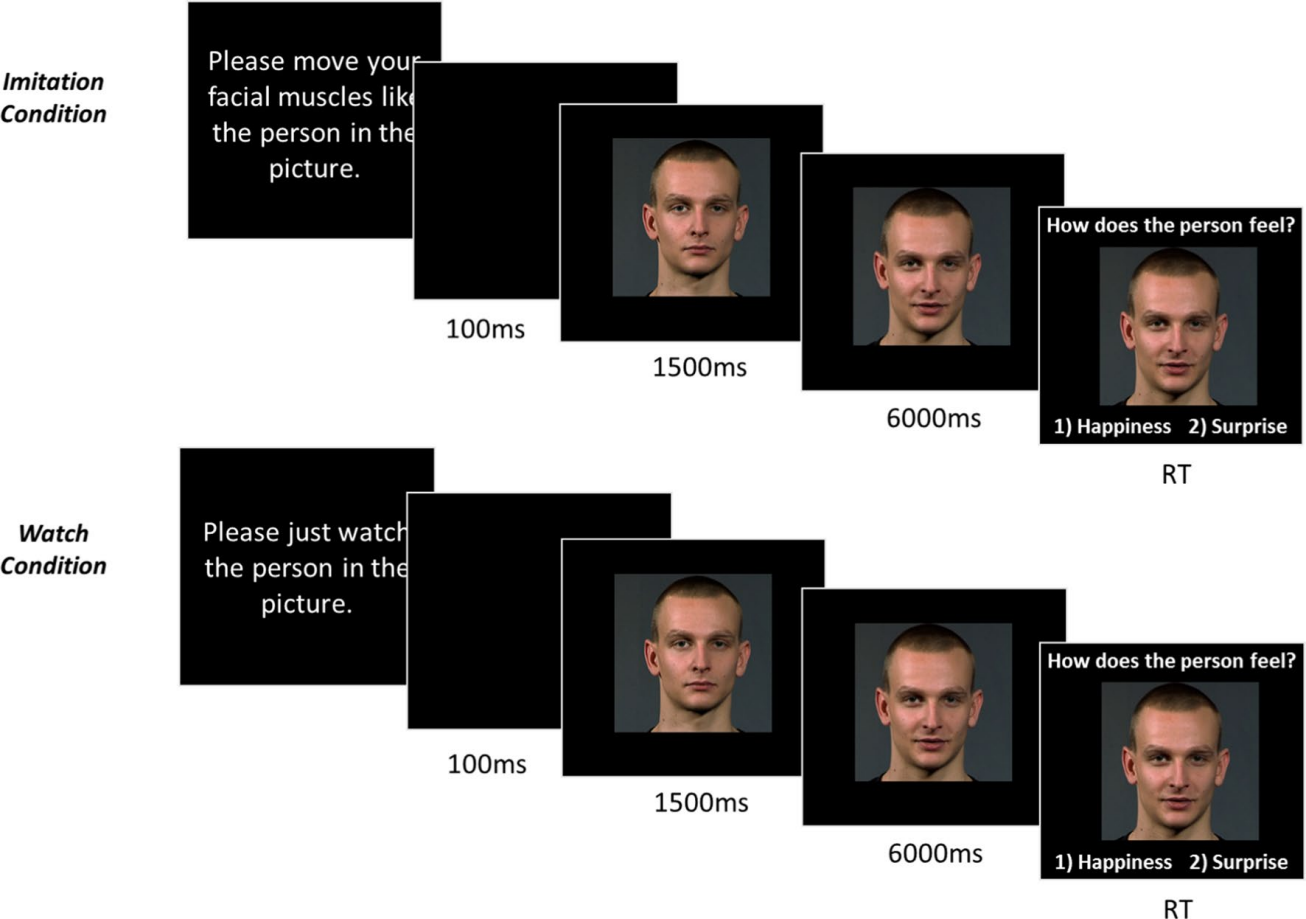
Time course of a trial of BERT for both conditions

To develop a sensitive task, the pictures were extracted from video clips in which professional actors expressed the target emotions. The actors had been instructed with emotional scripts (e.g. imagine you receive an unexpected present) to perform the facial expressions, starting with a neutral expression. This led to a more naturalistic footage. From each video clip, frames of three different intensities were extracted. These pictures of facial emotion expressions built the item pool for the BERT. This pool was reduced to the most sensitive items in a pre-study at a public open house event in Berlin, Germany, where large scientific institutions welcome the general public. In this pre-study with a sample of opportunity, 46 participants were asked to recognize the emotion of each of the items. Each picture was presented with the six basic emotions as possible answers. Based on their responses, for each video clip we selected the picture which discriminated best between low- and high-scoring participants. Additionally, we identified for each item the most difficult distractor out of the five incorrect emotion labels. In a follow-up online-study [37] with 436 participants, the selected pictures and distractors were tested and further improved with respect to reliability and discriminatory power by choosing the best eight items per emotion and most-difficult distractor. A more detailed description of the task development and the current version of the task can be found online under: http://www.hannadrimalla.de/bert.html.

#### Imitation

Each emotional expression was preceded by a picture of the same actor showing a neutral expression displayed for 1500 ms. This period was used as a baseline; thereafter the emotional expression picture was shown for 6 s before the emotional words appeared. During this period, the participant’s facial response to the picture was recorded via video, preventing movement artefacts resulting from behavioural responses. The reaction time was calculated, for correct answers only, from the moment when the emotion words appeared, and a response was made. After the participant’s reaction, the picture disappeared, a blank screen was shown for 100 ms, and the next trial began. Figure 2 displays the time course of a trial.

To investigate imitation, the BERT was presented in an imitation and a watch condition. In the imitation condition, the subject was instructed to move their facial muscles like the person in the photograph. The term “imitation” was not mentioned to mask the hypothesis. In the watch condition, the participant was instructed to just watch the person in the picture. Each condition consisted of 23 different pictures randomly drawn from the BERT picture pool. Due to a technical error, of the 48 pictures only 46 were randomly drawn for each participant. The order of the blocks was randomized.

#### Autistic traits

To assess autistic traits in both groups and to screen for ASC in the neurotypical group, the Autism-Spectrum Quotient [38] was administered in its German version [39].The AQ is a 50-item self-report questionnaire assessing different areas of behaviour and attitudes associated with autism spectrum conditions, such as social and communication skills, imagination and attention. On a 4-point scale, the participants indicate how strongly they agree or disagree with a statement. Every slight or strong agreement to an autistic behaviour adds one point to the total score. A score of 32 and above is seen as an indicator of autistic traits that might be clinically significant. The AQ has been shown to have good test–retest reliability and inter-rater reliability [38] as well as good discriminative validity and screening properties in clinical practice [40].

#### Automatic analysis of facial imitation behaviour

We chose a sign-based approach to measure participants’ facial expressions. Sign-based approaches are descriptive; they classify the visual input into abstract facial movements described by their location and intensity. As a coding system for these movements, the Facial Action Coding System (FACS [41]) is widely used in behavioural science and in automatic facial expression analysis. It breaks down facial expressions into 44 observable muscle movements, called action units (AU).

A major advantage of sign-based approaches is their objectivity as they do not involve interpretation [27]. Moreover, they do not reduce the complex emotional facial expression of a person to a small set of more abstract prototypical emotional expressions [42]. Last but not least, sign-based approaches allow us to preserve more dynamic information such as the time point, duration, and amplitude of an action [43]. This is crucial, as humans are very sensitive to the timing of facial actions [44]. Therefore, we chose a sign-based approach to measure participants’ facial expressions.

We employed the OpenFace 2.0. tool [45] to extract facial action units from the video recordings of the participant’s faces. OpenFace is an open-source tool capable of facial-landmark detection, head-pose estimation, facial-action-unit recognition and eye-gaze estimation. OpenFace 2.0 was trained on video data of people responding to an emotion-elicitation task. This corresponds to the conditions under which the BERT stimuli were recorded. Furthermore, it allows correcting for person-specific neutral expressions. OpenFace 2.0 has been tested on several emotion video data sets and demonstrated state-of-the-art results [45, 46].

OpenFace extracts the intensity (scale from 0 to 5) and the presence of 18 action units (AU) from each video frame (except for AU28, for which only presence is analysed). An overview of the AUs that can be detected by OpenFace is provided in Table 2 in “Appendix”.

To control for idiosyncrasies in the expression of the participant and their reaction to faces in general, we performed a baseline-correction. For each trial for each individual, we calculated the mean activity of each action unit during the baseline-phase (presentation of a neutral face). Next, for each trial we subtracted this baseline-activity from each action unit activity of each frame.

#### Measures of imitation

**Fig. 3:**
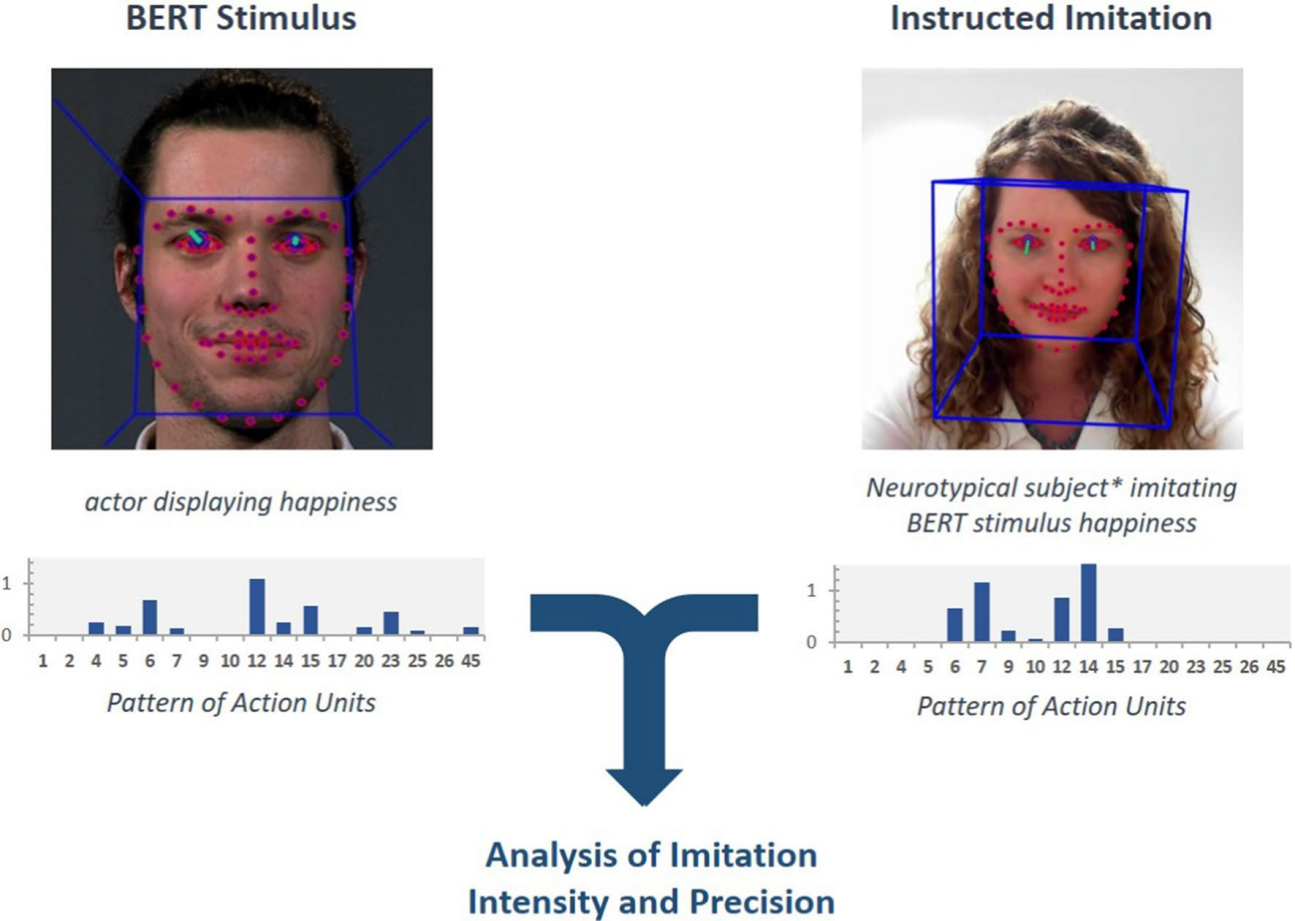
Automated facial analysis of imitation. At the left, the automated facial analysis of the stimulus material is displayed. At the right, the automated facial analysis of a neurotypical subject (*represented by the experimenter) in the imitation condition is displayed. Both measures are combined to analyze the imitation as described in the method section

**Fig. 4:**
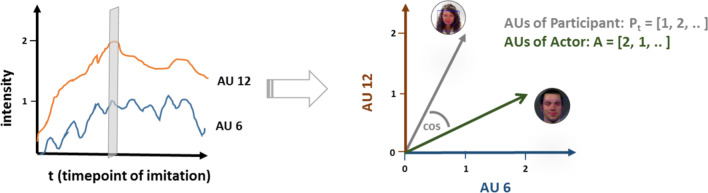
Calculation of cosine similarity between participant’s and actor’s vector

To assess the amount as well as the precision of the participant’s imitation (see Fig. 3 for a conceptual overview), we used two approaches: an imitation imprecision score (IIS) and cosine similarity measures (see Fig. 4).

The imitation imprecision score IIS indicates the *absolute* deviation of the participant’s facial expressions from the facial expressions displayed by the actors. Thus, it takes into account all AUs of the facial expression. Lower scores in IIS indicate a higher imitation precision. The imitation imprecision score IIS was calculated for each subject in two steps. First, we averaged the AU activity over frames, then averaged over AUs (Eq. 1), and finally we averaged over pictures (Eq. 2).

1 $${\text{IIS}}_{ps} = \mathop \sum \limits_{i = 1}^{a} \left( {\left| {\frac{{\mathop \sum \nolimits_{f = 1}^{{m_{ps} }} x_{{{\text{if}}_{ps} }} }}{{m_{ps} }} - \left( {x_{ip} } \right)_{{{\text{act}}}} } \right|} \right)$$

IIS_*ps*_, imitation imprecision score for picture *p* and subject *s*; *a*, total number of tracked action units; *m*_*ps*_, total number of frames for picture *p* for subject *s*; *x*_*if*_, intensity value of AU_*i*_ in frame *f*; *x*_*ip*_, intensity value of an AU_*i*_ shown in picture *p* by an actor *act*

2 $${\text{IIS}}_{s} = \frac{{\mathop \sum \nolimits_{p = 1}^{n} {\text{IIS}}_{ps} }}{n}$$

IIS_*s*_, action units-based imitation measure for each subject *s* averaged across *n* pictures; *n*, total number of pictures in a condition.

3 $$\hat{\mu } (\cos \left( \theta \right)_{s} ) = \frac{1}{m}*\mathop \sum \limits_{t = 1}^{m} \left( {\frac{{\mathop \sum \nolimits_{i = 1}^{n} P_{ti} \cdot A_{i} }}{{\sqrt {\mathop \sum \nolimits_{i = 1}^{n} P_{ti}^{2} } \cdot \sqrt {\mathop \sum \nolimits_{i = 1}^{n} A_{i}^{2} } }}} \right)$$

cosinus similarity averaged over all frames of trial *s*; *P*_ti_, participant’s action unit *i* intensity at timepoint *t*; *A*_i_, intensity value of actors’ action unit* i*; *t*, timepoint of imitation (frame); *n*, total number of action units; *m*, total number of frames of a trial.

For each frame, we calculated the cosine similarity of the actor’s and participant’s vector, which indicates whether the vectors point in the same direction, i.e. the expressions are similar (with 1 as highest possible value). We analyzed both the average as well as the maximum cosine similarity (highest value) of each trial for each participant. For the averaged cosine similarity, we first calculated the mean over all frames for a trial and then averaged over all trials of a participant. For the maximum cosine similarity, we calculated the maximum of all frames for a trial and then averaged the maxima over all trials of a participant.

To analyze the intensity of the imitation, we calculated the ratio of the participant’s vector’s length and the actress’ vector’s length at the time point of highest cosine similarity.

To analyze the speed of the imitation, we measured the time point (i.e. frame number) of maximum cosine similarity for each imitation of a participant. These 23 values were averaged for each participant.

#### Statistical analysis

In general, we used a significance level of *p* < 0.05. However, as we compared the imitation performance of the groups regarding four different aspects (imprecision, similarity, intensity and speed), we Bonferroni-corrected the level to a* = 0.0125 for these analyses. Data were analyzed using Python and R. In cases, in which we found evidence for a strong violation of the normal distribution assumption of our data, we used medians and nonparametric statistical tests indicated by the respective signs. Otherwise, we used means in combinations with parametric tests.

### Results

#### Demographics

The groups did not differ significantly with respect to age [*t*(78) = 1.77, *p* = 0.08], gender [*X*^2^(1, *N* = 80) = 1.10, *p* = 0.29], education (*W* = 765.5, *p* = 0.76) or verbal IQ [*t*(75) = 1.32, *p* = 0.19] as assessed through a German vocabulary test (Wortschatztest (WST), Schmidt Metzler [33]). For an overview of the demographic and diagnostic information of both groups, see Table 1.

As expected, the groups differed significantly regarding the AQ, with the mean AQ score being significantly higher in the ASC group than in the neurotypical group [ASC: *M* = 37.54, SD = 5.74; control group: *M* = 14.49, SD = 5.82; *t*(74) = 17.33, *p* < 0.001]. No participants from the neurotypical group scored above the cut-off score of 32.

### Emotion recognition

#### Reliability and item analysis of BERT

Internal consistency of the BERT was assessed with Cronbach’s alpha and McDonald’s omega. Item difficulty was defined as the mean percentage of correct responses over all participants divided by the number of all participants. The following satisfactory results were obtained: *n* = 80, Cronbach’s *α* = 0.74, McDonald’s *ω*_*T*_ = 0.75, mean item difficulty = 0.77, range = 0.36 − 0.99.

#### Effects of autism diagnosis

Across both conditions of the emotion classification task, the NT group showed a higher percentage of correct emotion classification than the ASC group [NT: 79%; ASC: 73%; *t*(78) = 2.96; *p* = 0.004, *d* = 0.67] and faster responses (ASC: 4100 ms, NT: 2832 ms; *z* = 395, *p* < 0.001, *r* = 44.16). We calculated two mixed effects regression models regarding the emotion recognition abilities of the participants. The first model was built to predict the percentage of correct responses with group and imitation-instruction as fixed effects and a random intercept for each participant. The second model aimed to predict the reaction times of correct responses with group and imitation-instruction as fixed effects and a random intercept for each participant. Both models supported lower emotion recognition of the individuals with ASC, for the correct responses (*β* =  − 0.054; 95% CI [− 0.093, − 0.015]; *z* =  − 2.73, *p* = 0.006) as well as for the reaction times (*β* = 0.34; 95% CI [0.201, 0.470]; *z* = 4.89, *p* < 0.001).

### Facial imitation

Four participants who, irrespective of imitation condition, imitated the facial expression either never or always, were excluded. Thus, the analysis of imitation effects was calculated on 41 neurotypical subjects and 35 individuals with ASC. Further, for the analysis of the facial expression movements, four participants were excluded, as tracking in these cases was flawed. The resulting sample size for each group was 39 neurotypical individuals and 33 individuals with ASC. We used linear mixed-effects models to control for individual differences and deal with missing values. We built a model with the fixed factors autism diagnosis, imitation-instruction and their interaction. Additionally, we modelled a random intercept and a random slope for each participant to control for random individual baseline differences and differences in their reaction to the mimicry instruction. We aggregated the data for each participant separately for the two conditions.

Due to the novel use of computational approaches in facial expression analysis and due to a lack of literature, it is unclear if such approaches are sensitive enough to detect even very small muscle movements, as is the case for spontaneous mimicry. Thus, for this study, we focused on the facial expressions in the instructed imitation condition only, for which we expected marked facial movements.

We focused on four measures of imitation performance: the imprecision (IIS, absolute difference of actor’s and participant’s AUs), the similarity of their expression (cosine similarity of their AU vectors), the intensity of their most similar expression (ratio of their vector’s length at the time point of highest cosine similarity) and the speed of the imitation. First, we checked whether both groups were able to imitate, i.e. showed a higher mean and maximum similarity in the imitation than in the watch condition. Second, we compared individuals with and without autism on all imitation measures. Third, for the individuals with autism we analyzed whether these measures were associated with their level of symptoms on the social domain indicated by the respective ADOS subscore.

### Comparison of imitation and watch condition (separated by groups)

#### Cosine similarity of imitation in NT individuals

The neurotypical participants successfully imitated the expression. If they were instructed to imitate, they displayed expressions that were significantly more similar to the target expression (measured by cosine similarity averaged across time and all pictures) compared to when they just watched the expressions [difference: *M* = 0.063, 95% CI [0.06 0.10], *t*(38) = 8.57, *p* < 0.001, *d* = 1.39], as well as compared to the baseline condition [difference: *M* = 0.38, 95% CI [0.37 0.39], *t*(38) = , *p* < 0.001, *d* = 10.44)]. In line with this finding, the maximum cosine similarity, i.e. most similar expression during the complete trial, was also higher in the imitation condition (difference: *Mdn* = 0.076, *Z* = 71, *p* < 0.001).

#### Cosine similarity of imitation in individuals with ASC

Individuals with autism also imitated the presented facial expressions when they were instructed to. In the imitation condition, they displayed expressions that were significantly more similar to the target expression (measured by cosine similarity averaged across time and all pictures) than towards the watch condition (difference: *M* = 0.07, 95% CI [0.05 0.09], *t*(32) = 8.65, *p* < 0.001, *d* = 1.53), as well as compared to the baseline condition (difference: *M* = 0.38, 95% CI [0.36 0.40], *t*(32) = , *p* < 0.001, *d* = 8.14) In line with this finding, the maximum cosine similarity, i.e. most similar expression during the complete trial, was also higher in the imitation condition (difference: *Mdn* = 0.086, *Z* = 36, *p* < 0.001).

### Group comparison of individuals with and without ASC (imitation condition)

#### Similarity of Imitation

In accordance, we found no evidence that individuals with autism showed less similar expressions, averaged over all pictures, than neurotypical individuals, neither regarding the mean similarity [*M*_ASC_ = 0.38, *M*_NT_ = 0.38, *t*(70) = 0.14, *p* = 0.890, *d* = 0.033] nor the maximum expressed similarity (*Mdn*_ASC_ = 0.71, *Mdn*_NT_ = 0.69, *Z* = 585, *p* = 0.256).

#### Intensity of imitation

**Fig. 5:**
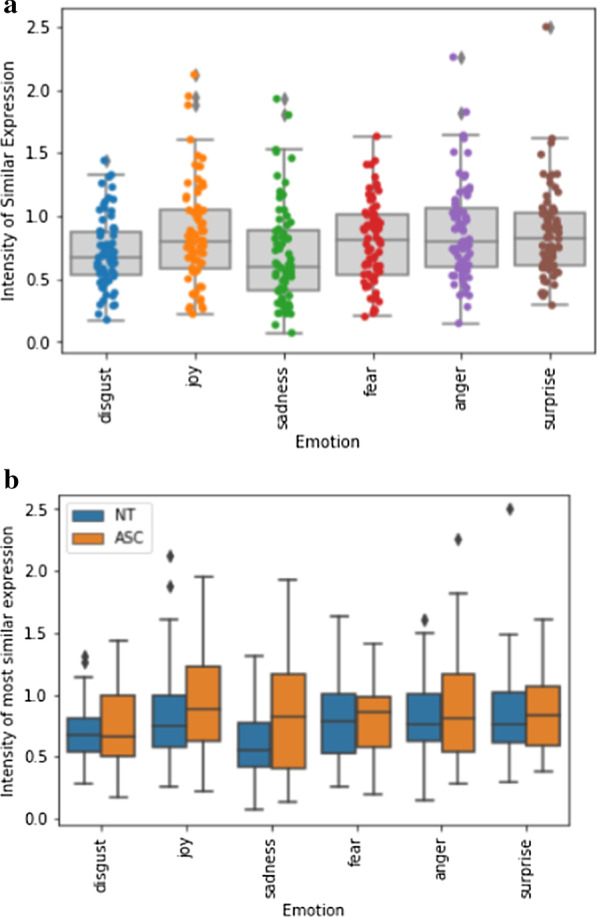
**a **Comparison of intensity of most similar expression in the imitation condition separated by emotions. **b** Comparison of intensity of most similar expression in the imitation condition for neurotypical individuals (left) and individuals with autism (right) separated by emotions

Further*,* we found no evidence that individuals with autism showed less intensity of maximal similarity (*M*_ASC_ = 0.85, *M*_NT_ = 0.77, *t*(70) = 1.45, *p* = 0.153*, d* = 0.347). Figure 5a shows the intensity of the most similar expression averaged over trials for each participant and separated by emotion categories, and Fig. 5b shows the intensity of the most similar expression averaged over trials for both groups and separated by emotion categories.

#### Imprecision of imitation

We measured the precision of the imitated expression by calculating the difference from the original stimuli (IIS_*s*_). The facial expressions that individuals with autism showed differed from the actors’ expression (*Mdn* = 8.90) significantly more than the facial expressions of the neurotypical individuals differed from the actors’ expression (*Mdn* = 8.50), *U* = 444, *p* = 0.012, *d* = 0.3.

##### Post Hoc: Variance of Imitation in Individuals with ASC

**Fig. 6:**
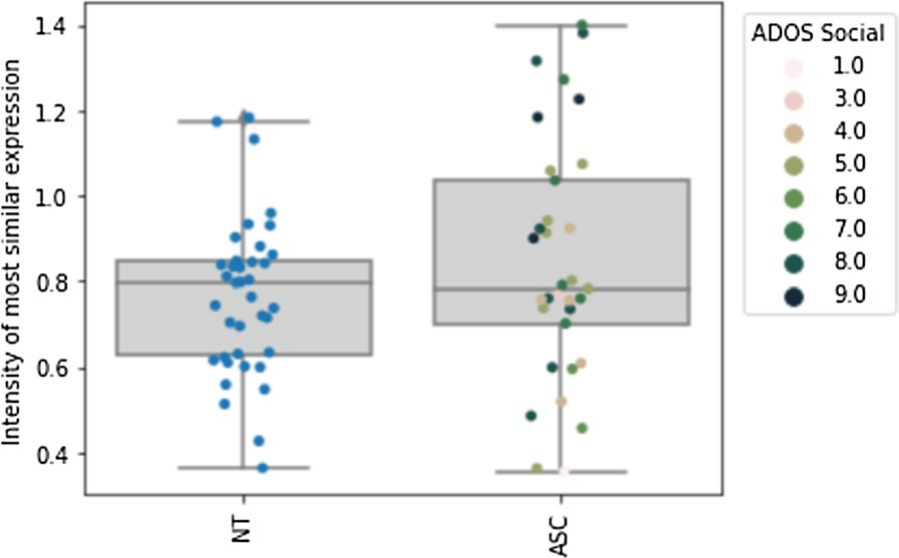
Comparison of intensity of most similar expression in the imitation condition for neurotypical individuals (left) and individuals with autism (right)

Post hoc, we compared the variance of the imitation measures between both groups. There was significantly more variance in the group of individuals with autism than in the neurotypical group regarding the intensity of the imitation (*F* = 4.63, *p* = 0.035). The distribution of imitation intensity can be seen in Fig. 6. Further, there was a tendency to more variance regarding the maximum similarity (*p* = 0.096).

##### Timing of Imitation in Autism

**Fig. 7:**
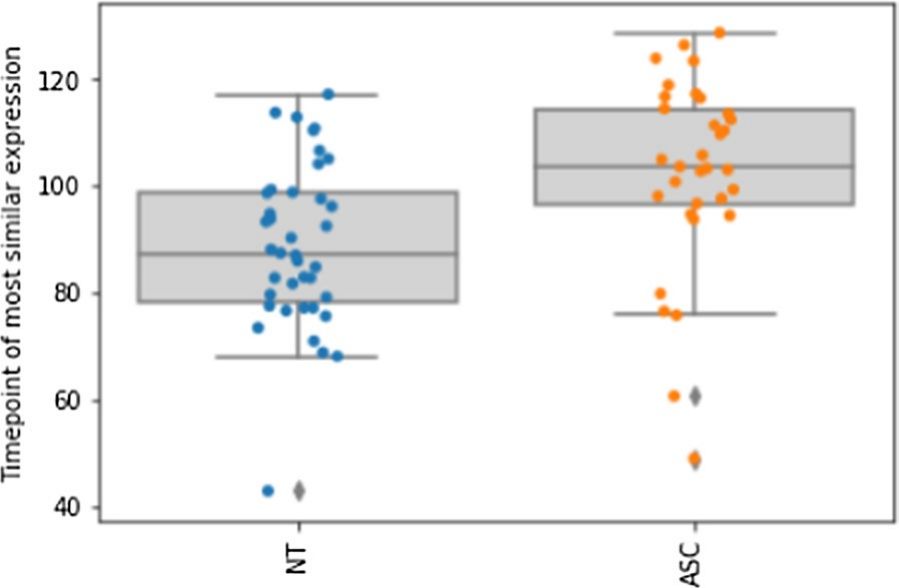
Comparison of timepoint (i.e. number of frames) of maxima of relevant action units during imitation for neurotypical individuals (left) and individuals with autism (right)

To analyze the speed of the imitation, we measured the time point (i.e. frame number) of maximum cosine similarity. Individuals with autism needed significantly more time (*M* = 102.56 95%CI [96.17 108.95]) than neurotypical individuals (*M* = 88.90 95%CI [83.95 93.84]) for the imitation, *t*(70) = − 3.48, *p* < 0.001, *d* = 0.84. In general, the speed of the imitation scaled positively with the speed of the emotion recognition of an individual (*r* = 0.388, *p* < 0.001). The participant’s averaged time for maximum imitation can be seen in Fig. 7 for both groups.

#### Dimensional Relationship of social ADOS and Imitation

Severity of autism social symptomatology (social ADOS) was positively associated with both the maximum intensity of the imitation (*r* = 0.445, *p* = 0.009) and negatively associated with the similarity of imitation and target expression (*r* = − 0.477, *p* = 0.005) and the maximum similarity (*r* = − 0.512, *p* = 0.0023). Further, severity of social autism symptomatology (social ADOS) correlated positively with the imprecision of the imitation (IIS;* r* = 0.357, *p* = 0.041) in autistic individuals, which, however, did not survive Bonferroni-correction.

### Emotion recognition and imitation

#### Effects of instructed imitation on emotion recognition

Imitation influenced emotion recognition performance negatively: under the condition of imitation, both participant groups showed lower rates of correct responses (*β* =  − 0.03; 95% CI [− 0.061, − 0.001]; *z* =  − 2.03, *p* = 0.042) and needed more time for the recognition (*β* = 0.05; 95% CI [0.015, 0.087]; *z* = 2.79, *p* = 0.005). There was no evidence for an interaction effect of autism and imitation if those were included as additional factor (for correctness *p* = 0.814 and for response time *p* = 0.379).

#### Effects of amount of imitation on emotion recognition

To further investigate the relationship between imitation of an expression and emotion recognition performance, we calculated a model separately for the imitation condition. For the precision and occurrence of the imitation, we calculated a regression model that controlled for the group as well as for the interaction. We calculated an Ordinary Least Squares regression predicting the percentage of correct answers and using the IIS and the autism condition as predictors. The accuracy of the emotion recognition scaled negatively with the individual imprecision of imitation (*β* = − 0.039; 95% CI [− 0.068 − 0.010]; *z* = − 2.67, *p* = 0.009). We found no effect on the speed of correct answers (*β* = 75.90, *p* = 0.706). Further, we found no relationship between emotion recognition and the cosine similarity measures (all *p* > 0.05).

## Discussion

Based on a large sample and computer-based facial analysis, we measured quantitative and qualitative differences in facial imitation as well as emotion recognition between individuals with and without autism. Both groups showed intact imitation of facial expressions when they were instructed to imitate. However, the group of individuals with autism differed from neurotypical individuals regarding the speed and precision of the imitation: their voluntary imitation was on average slower and less precise. A separate analysis of the imitation’s intensity and its similarity with the actor’s facial expression revealed an association between these measures and the severity of the social deficits in the individuals with autism. The more affected individuals expressed a less similar but more intense imitation.

On average, individuals with autism recognized fewer emotions correctly and were slower in imitating them than neurotypical individuals. While the effect was small for recognition accuracy, it was of greater magnitude for recognition time. For both groups, the instruction to imitate the emotional expressions was associated with decreased performance in emotion recognition compared to the watch condition. However, the precision of the imitation was positively associated with recognition performance across groups.

### Group differences in emotion recognition

We replicated previously reported difficulties of individuals with autism to recognize emotions from facial expressions (e.g. [36, 47, 48]). Using expressions of basic emotions with varying intensities, which were designed to be difficult to interpret and thus more sensitive, we were able to show that even high-functioning adults with autism seem to have difficulties in recognizing them, in that they need more time and make slightly more errors. These results are consistent with previous studies on emotion recognition in individuals with ASC, that report differences in reference to emotion recognition of briefly presented stimuli [49, 50]. In real life, emotions often occur briefly in a subtle form comparable to our stimulus material, which might, at least partially, explain the social difficulties of high-functioning individuals with autism in daily life [15].

### Group differences in facial imitation

In accordance with similar studies, we found that individuals with autism were on average capable of imitating facial expressions when instructed (e.g. [18, 19, 23]).

Using computer-based face analysis, we could also measure and quantify qualitative differences in imitation, especially in dependence of the level of autism symptoms in the social domain. Most studies so far have focused on the occurrence of facial imitation and ignored qualitative differences. Preliminary descriptions of such qualitative differences exist, e.g. Loveland et al. [22] stated that “the responses of subjects with autism contained many unusual behaviours, such as bizarre expressions and those that looked ‘mechanical’”. Another study, which measured the spontaneous and instructed imitation of facial expression in children with autism, pointed to an altered time course of the facial expressions in individuals with ASC, but only for spontaneous imitation [23].

We replicated this timing effect for imitation, as we found that individuals with autism needed on average more time than neurotypical individuals to imitate facial expressions voluntarily. As dynamic properties of facial expression (e.g. time point of maximal expression, duration, etc.) play an important role for the perceived genuineness [51], the group differences in timing might in part underlie the social interactions problems that individuals with autism show. Further, the imitation speed was associated with the recognition speed. This might point to the importance of imitation for emotion recognition. However, it could also be interpreted as generally slower processing times, which underlie both imitation and recognition. Although intelligence might further represent a factor underlying this association, it is less likely to play a role here, given that our participant groups were matched for IQ. In addition to the timing of facial expressions, individuals with autism differed on average from neurotypical individuals regarding the mean precision of their imitation of emotional expressions. This matches a finding by Brewer et al. [52], which reported that posed facial expressions of emotions of individuals with autism were recognized less accurately than of neurotypical individuals by both individuals with and without autism.

Further, the imitation quality of the individuals with autism was associated on average with their ADOS social subscores, evident in three different measures of imitation performance (similarity, intensity and, marginally, imprecision). In accordance with this finding, Yoshimura et al. [53] showed an association between facial imitation extent and social functioning. Partially, our finding regarding the negative association between imitation performance and severity of autism resonates with a study of Faso and colleagues [54] as well. The authors compared posed and evoked facial expressions of adults with and without ASC. Naive observers rated the expression of individuals with ASC as more intense and less natural. However, we did not replicate this difference regarding similarity and intensity of the imitation on a group level, presumably because of a less affected patient group. A post hoc analysis, supports this interpretation, as there was more variance in the group of individuals with autism than in the neurotypical group regarding the intensity of their imitation. Second, the negative association of imitation performance and social symptomology without a group-difference in contrast to the neurotypical individuals might be explained by the heterogeneity within the autism population, especially as some individuals predominantly show impairments in one of the two domains, either the social communication and interaction domain or the domain of repetitive behaviour [55]. Thus, a clear group difference regarding the imitation performance might only be evident if individuals with social deficits are compared with neurotypical participants. This interpretation is in accordance with the results of a recent study of Zane et al. [56], which compared facial expressions of neurotypical individuals and individuals with autism in an instructed imitation of emotional expressions task. Similar to our study, the authors found more variance regarding the intensity of their facial expressions in the group of individuals with autism compared to the neurotypical group.

The difficulties of, especially more severely affected, individuals with ASC in generating facial expressions might be associated with their lower tendency to engage in impression management such as in displaying social laughter [57–59]. One possible reason might be a reduced motivation of individuals with ASC for social maintaining [60]; another might be a reduced ability to finetune one’s facial expressions. The second explanation corroborates recent evidence that individuals with ASC show less precise imitation regarding hand movements [61, 62]. These findings favour the assumption that individuals with ASC demonstrate difficulties in the finetuning of imitation [63] rather than an inability to imitate [64].

Our findings also match at least partially the summary of a recent meta-analysis [24] investigating facial expression production in autism. Trevisan and colleagues concluded that participants with ASC display facial expressions less frequently, for a shorter amount of time and less accurately. Further, they stated that individuals with ASC do not express emotions less intensely nor slower. As explained above, the null effect regarding intensity might be explained by not considering the level of social impairments. In general, the comparison of our results with this meta-analysis should be taken with caution, as the meta-analysis covers a high number of very different studies including some on spontaneous expressions, mimicry and verbally prompted posing of facial expression.

### Relationship of imitation and recognition of facial emotions

Comparing the imitation condition to the watch condition revealed a negative effect of the instruction to imitate on emotion recognition performance across groups. This finding is consistent with that of Kulesza et al. [65], who asked healthy participants to recognize basic emotional facial expressions of an actress and found that participants who were instructed to imitate the expression recognized less facial displays of the emotions than participants who were instructed to inhibit spontaneous imitation of the expressions. In accordance with these findings, in a study with healthy individuals by Stel et al. [66], mimicking facial and behavioural movements of an interaction partner reduced another aspect of emotional understanding, i.e. detecting whether the partner was lying.

That being said, those results cannot rule out the possibility that imitation does foster emotion recognition after all. For example, another possible reason for the negative effect of the imitation on the accuracy of emotion recognition in our design is an additional cognitive load. Controlling the facial muscles might absorb cognitive energy in the imitation condition, and thereby worsen emotion recognition. In line with this interpretation are the results of a study by Lewis et al. [67]. The participants in this study performed an emotion recognition task twice, and half of the participants had to imitate the facial expression in the second round. As both groups recognized more emotions in the second round, it can be assumed that the participants’ cognitive load for the emotion recognition task itself was reduced in the second round. While mimicking did not help the performance at the baseline test, the increase in performance in the second round was significantly higher for the mimickers. It seems plausible that only the lower cognitive load in the repeated condition allowed mimicry to take an effect. Thus, individuals’ emotion recognition might benefit from imitation, if the imitation does not involve much extra cognitive load. In our design, all stimuli were presented only once, resulting in two equally difficult conditions. This might overshadow any possible positive effect of imitation.

That beneficial effects of imitation on emotion recognition might indeed exist is indicated by our finding that the intensity as well as the precision of imitation was positively associated with emotion recognition performance across the whole group of participants. However, given the correlational nature of this finding, the interpretation warrants caution as the finding could also be explained in a way that either recognition of an emotion mediates imitation or that the severity of autism social symptoms act as a confounding variable.

Most studies investigating facial expressions in autism suffer from low statistical power and might be biased by low intellectual level or age of the participants (for an overview, see [24]). We managed to collect a study sample of 80 individuals, including 37 individuals with autism and without intellectual impairment and ensured an equal portion of male and female participants. A further strength of this study is its unobtrusive measure of facial expressions, which is particularly relevant for individuals with autism and allowed us to research a large sample of this population.

### Limitations

As our study investigates adults with autism spectrum condition without intellectual impairment, we do not know whether our findings hold for children with autism or adults with intellectual impairment. A further limitation of this study is its unknown sensitivity to measure non-observable imitation, as OpenFace only assesses muscle movements detectable by camera, whereas EMG allows assessment of very subtle muscle movements [68]. However, OpenFace 2.0. and its precursor OpenFace toolkit have shown their usefulness in studies, which aimed to detect suicidal ideation [69], psychotic symptoms [70] and autism [28] based on facial expressions, which speaks for its general sensitivity. Another potential further limitation of our study is that we cannot rule out that participants moved their facial muscles voluntarily in the watch condition. However, the negative results for imitative behaviour in that condition speak against this having occurred. Additionally, individuals with gross voluntary movement during the watch condition were excluded based on the individual screening of all video recordings.

In our analysis, we first applied a general baseline-correction to correct for the participant’s general facial expression. Second, we calculated a trial-wise baseline-correction to measure the participant’s imitation in comparison with their reaction to the actor’s neutral face. As a result, our imitation measures are measures of change and movement of someone’s face. This baseline correction implies, however, that a person that shows a certain emotional expression during the neutral phases of the experiment (e.g. because she feels anxious throughout the experiment) might receive a lower imitation score for showing a similar emotional expression as the person to be imitated. However, we are not interested in the absolute facial expression but in the change towards someone’s neutral face. This change, from a neutral baseline expression to a more emotion-specific expression, significantly occurred in both groups, evident as a higher cosine similarity averaged above all six basic emotions and participants. Due to a technical error, not all participants saw the same stimuli. However, as the differences were very small and by random choice, we do not assume that this effected our results.

Aiming at a voluntary imitation condition that would be as clearly defined as possible, while not necessitating explicit emotion processing, we asked the participants to “move their facial muscles like the person in the photo”. We avoided mentioning the term “imitation”, as it might activate popular science beliefs about imitation and its effects on emotion recognition. However, people might scan faces differently if the instruction creates an explicit focus on the muscles rather than the emotion, e.g. by looking less to the eyes and more at other parts of the face. It is also possible that NT and ASC groups respond to this instruction differently, with ASC potentially focusing more literally on muscles rather than the holistic emotion expression. Further studies including eye-tracking should elucidate this aspect as well as the process of imitation more fine-grained.

Aiming for a high standardization, we explicitly asked participants to imitate a static expression display in a photograph for a specific time, instead of collecting facial imitation in the wild. The aim of the study was to investigate the general ability of individuals with autism to imitate facial expressions if there are instructed to. In social interactions, emotions are sometimes expressed voluntarily to produce a certain impression or present oneself in a socially desirable way [71]. However, the results need to be interpreted with caution, as it is not clear, whether people would behave differently in the real-world, e.g. due to different contexts, additional load, the dynamics of facial expressions or social motivations. Further, it has been shown that voluntary imitation relies on different underlying processes than spontaneous imitation [72]. Thus, it would be of great value to conduct a similar study in a real-world setting to see if the results generalize to those settings. In such an experiment, computer-based measures may help to enable an unobtrusive measurement of facial expression imitation. Still, as previous research has often claimed that voluntary facial imitation is not affected in individuals with autism [18], we consider it important to elucidate these differences in our work.

Indeed it is important to bear in mind that our understanding of how facial expressions are used in the real world is still very limited [73]. Further research is needed to better understand how people move their faces in different contexts of everyday life and how they use their facial movements to transfer social information.

Finally, yet importantly, the positive relationship of emotion recognition and imitation extent and precision could only be shown as a correlation. Further studies are needed to investigate the causal direction of this relationship.

## Conclusions

To the best of our knowledge, this is the first study that successfully used computer-based analysis to measure facial expression in an imitation context. This unobtrusive and affordable method allowed us to measure qualitative differences in facial expressions between neurotypical individuals and individuals with autism. Using the newly developed sensitive emotion recognition task BERT, we were able to replicate the emotion recognition deficit in individuals with autism and provided some evidence for a positive association of imitation performance and the recognition of emotions.

Further research should explore facial expressions in social interactions with active and passive roles of the participants (expressing and recognizing emotions) to exclude the artificial load of the instruction to express an emotion in imitation paradigms. More broadly, research is also needed to determine the potential of training imitation as a possible mechanism to enhance emotion recognition. While imitation does not seem to help emotion recognition immediately (likely due to additional task demands), training imitation precision via instruction might enhance spontaneous imitation and by that foster emotion recognition.

## Data Availability

The datasets generated and analyzed during the current study are not publicly available due to privacy restrictions of the video data but are available from the corresponding author on reasonable request. The BERT [34] is available online under GNU General Public License Version 3.0 from http://www.hannadrimalla.de/bert.html.

## Appendix

**Table 2:** Selected single action units from the Facial Action Coding System (FACS) [41]; table adapted from Rosenberg [75] (AU45, which refers to an eye blink, is not listed here but detected by OpenFace)

AU number	FACS name	Muscles
AU01	Inner Brow Raiser	Frontalis, Pars Medialis
AU02	Outer Brow Raiser	Frontalis, Pars Lateralis
AU04	Brow Lowerer	Depressor Glabellae; Depressor Supercilli, Corrugator
AU05	Upper Lid Raiser	Levator Palpebrae Superioris
AU06	Cheek Raiser	Orbicularis Oculi, Pars Orbitalis
AU07	Lid Tightener	Orbicularis Oculi, Pars Palebralis
AU09	Nose Wrinkler	Levator Labii Superioris, Alaeque Nasi
AU10	Upper Lip Raiser	Levator Labii Superioris, Caput Infraorbitalis
AU11*	Nasolabial Furrow Deepener	Zygomanic Minor
AU12	Lip Corner Puller	Zygomatic Major
AU14**	Dimpler	Buccinator
AU15	Lip Corner Depressor	Triangularis
AU16*	Lower Lip Depressor	Depressor Labii
AU17	Chin Raiser	Mentalis
AU20	Lip Stretcher	Risorius
AU22*	Lip Funneler	Orbicularis Oris
AU23	Lip Tightener	Orbicularis Oris
AU24*	Lip Pressor	Orbicularis Oris
AU25	Lips Part	Depressor Labii, or Relaxation of Mentalis or Orbicularis Oris
AU26	Jaw Drop	Masetter; Temporal and Internal Pterygoid Relaxed
AU27*	Mouth Stretch	Pterygoids; Digastric
AU28**	Lip Suck	Orbicularis Oris

*An AU cannot be detected by Open Face [45]

**An AU can be tracked by OpenFace but is not considered relevant for basic emotions in FACS [41], as cited by Gosselin, Kirouac and Doré [74]

## Abbreviations

ADI-R: Autism Diagnostic Interview-Revised.
ADOS-2: Autism Diagnostic Observation Schedule 2
AQ: Autism Spectrum Quotient
ASC: Autism Spectrum Condition
AU: Action Unit
BERT: Berlin Emotion Recognition Test
IIS: Imitation Imprecision Score
WST: Wortschatztest
